# A History of the Discovery of the Circulation of the Blood

**Published:** 1859-09

**Authors:** 


Art. IV.—*A History of the Discovery of the Circulation of the Blood.* By P. Flourens, Perpetual Secretary of the Academy of Sciences, (Institute of France;) Professor of the Museum of Natural History of Paris, etc. etc. Translated from the French, by J. C. Reeve, M.D. pp. 175. Cincinnati: Rickey, Mallory & Co. 1859.

In a former number of this journal we gave our readers a somewhat extended review of this work, for the reason that no English translation had at that time been made. We are now gratified in being able to announce an American edition just issued from the press, in a style that reflects much credit upon the publishing enterprise of the West.

In the preface to the edition before us the translator justly remarks that

"This discovery may be said, without exaggeration, to be one of the most important events in the history of medical science; some of the greatest minds ever in the profession took part in it; it exerted an important influence upon the treatment of disease; it marked a new era in the history of medicine, and effected a revolution in scientific research."

Dr. Reeve is an accomplished French scholar, and in furnishing a faithful translation of what may safely be said to be the most complete history of the discovery of the circulation of the blood that has ever been published in the English language, he has supplied information upon a most interesting topic, and furnished a neat and most readable little volume that should be found in every medical library.

